# Anti‐tumorigenic effects of naive and TLR4‐primed adipose‐derived mesenchymal stem cells on pancreatic ductal adenocarcinoma cells

**DOI:** 10.1002/cam4.6964

**Published:** 2024-02-01

**Authors:** Demet Kaçaroğlu, Seher Yaylacı, Nilgun Gurbuz

**Affiliations:** ^1^ Department of Medical Biology, Faculty of Medicine Suleyman Demirel University Isparta Turkey; ^2^ Department of Medical Biology, Faculty of Medicine Lokman Hekim University Ankara Turkey

**Keywords:** mesenchymal stem cells, MSC1, pancreatic cancer, TLR4, tumor microenvironment

## Abstract

**Background:**

One of the main reasons for the unsuccessful treatment of pancreatic cancer is the intense desmoplastic pancreatic microenvironment. In the literature, the effects of mesenchymal stem cells (MSCs) and their inflammatory phenotypes on cancer cells have been a subject of controversy. Therefore, it is crucial to elucidate the underlying mechanisms of this interaction, especially in the context of pancreatic cancer. We aimed to investigate the effects of naive, TLR4‐activated, and TLR4‐inhibited phenotypes of adipose‐derived MSCs (ADMSC) on pancreatic ductal cell line (Panc‐1).

**Methods and Materials:**

Adipose‐derived MSCs were induced into a proinflammatory phenotype using a 0.5 μg/mL dose of TLR4 agonist, while an anti‐inflammatory phenotype was generated in ADMSCs using a 25 μg/mL dose of TLR4 antagonist. We observed that the proliferation of Panc‐1 cells was inhibited when naive ADMSCs:Panc‐1(10:1) and proinflammatory ADMSCs:Panc‐1(10:1) were directly cocultured.

**Results:**

In indirect coculture, both naive and proinflammatory ADMSCs exhibited a significant 10‐fold increase in their inhibitory effect on the proliferation and colony forming capacity of Panc‐1 cells, with the added benefit of inducing apoptosis. In our study, both naive and proinflammatory ADMSCs were found to regulate the expression of genes associated with metastasis (MMP2, KDR, MMP9, TIMP1, IGF2R, and COL1A1) and EMT (CDH1, VIM, ZEB1, and CLDN1) in Panc‐1 cells. Remarkably, both naive and proinflammatory ADMSCs demonstrated antitumor effects on Panc‐1 cells. However, it was observed that anti‐inflammatory ADMSCs showed tumor‐promoting effects instead. Furthermore, we observed a reciprocal influence between ADMSCs and Panc‐1 cells on each other's proinflammatory cytokine expressions, suggesting a dynamic interplay within the tumor microenvironment.

**Conclusions:**

These findings underscore the significance of both the naive state and different inflammatory phenotypes of MSCs in the microenvironment and represent a pivotal step toward the development of novel therapeutic approaches for pancreatic cancer. Understanding the intricate interactions between MSCs and cancer cells may open new avenues for targeted interventions in cancer therapy.

## Graphical abstract

In our study, we showed that the tumorigenic effects of ADMSCs on Panc‐1 cells were altered when stimulated via TLR4 signaling, but this effect was altered when naive ADMSCs and ADMSCs with pro‐inflammatory character stimulated by TLR4 agonist exerted anticarcinogenic effects on Panc‐1 cells. Anti‐inflammatory ADMSCs, on the other hand, showed tumor‐promoting effects. Understanding the role of MSCs in the tumor microenvironment will be a guiding factor for the development of microenvironment‐targeted therapeutic approaches in the future.

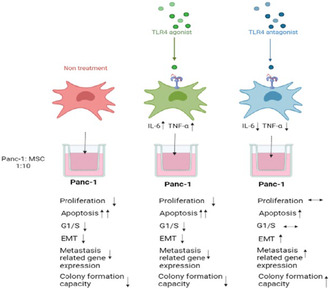

## 1 INTRODUCTION

Pancreatic ductal adenocarcinoma (PDAC) represents approximately 90% of all cases. Tragically, current treatment approaches for pancreatic cancer are inadequate, resulting in a low survival rate.^1^ Pancreatic cancer presents nonspecific symptoms and the lack of specific markers leads to delayed diagnosis of pancreatic cancer.^2^ Treatment options for pancreatic cancer include surgical resection, radiation therapy, chemotherapy, immunotherapy, or combinations thereof, depending on the stage of the disease. However, pancreatic cancer is known to be resistant to these conventional treatments.^3^

Pancreatic ductal adenocarcinoma is characterized by the simultaneous disruption of multiple cellular mechanisms at the molecular level, including mutations in tumor suppressor genes, overexpression of oncogenes, somatic point mutations, allelic loss, excessive activation of proliferative signaling pathways, and mutations in genes controlling the cell cycle.^4^ The fact that 91% of PDAC patients are diagnosed with a high risk of metastasis. Given the rapid occurrence of metastasis in PDAC, understanding the fundamental processes that drive metastatic behavior is of critical importance in determining how to approach the disease in a clinical setting.^5^ Recent studies have demonstrated that the microenvironment plays a crucial role in PDAC progression, revealing a close relationship between the microenvironment and metastasis.^6^, ^7^ In PDAC, approximately 90% of the tumor mass is composed of desmoplastic stroma. Moreover, this microenvironment is not static and plays a role in promoting the aggressiveness of tumor cells, despite constituting only 10% of the total tumor cell population.^8^ Overall, this desmoplastic microenvironment in pancreatic cancer creates a supportive environment for tumor cells, promoting their proliferation, invasion, metastasis, angiogenesis, altered metabolism, immune suppression, and development of chemoresistance.^6^ Mesenchymal stem cells (MSCs), as a form of cancer‐associated fibroblast, are one of the most important regulatory cells in the pancreatic tumor microenvironment.^7^ To be able to intervene in the tumor microenvironment, it is important to first elucidate the interactions between MSCs and tumor cells.

The effects of MSCs, which are among the cells that make up the stroma, on tumor cells and therapeutic approaches related to them are a current research topic. Initially, when MSCs first arrive at the tumor site, they exhibit inhibitory activity against the tumor. However, over time, they polarize and exhibit tumor‐promoting activity based on signals received from the cancer cells. Mesenchymal stem cells can transform into cancer‐associated fibroblasts (CAFs) and support tumor cell proliferation, angiogenesis, evasion of apoptosis, development of chemoresistance, and metastasis.^9^ Indeed, when naive MSC first arrive in the tumor microenvironment, they exhibit a pro‐inflammatory and anti‐tumor phenotype. However, over time, based on signals received from the surrounding environment, they can transition into an anti‐inflammatory and pro‐tumorigenic character. This phenotypic switch is influenced by various factors and signaling molecules present in the tumor microenvironment. The transformation of MSCs into a protumorigenic phenotype can contribute to tumor progression and the development of a supportive environment for tumor growth and survival.^10^ Mesenchymal stem cells affect all cells in the same microenvironment by secreting cytokines or by direct contact with receptors. In vitro and in vivo research has shown that MSCs exhibit both tumor‐promoting and antitumorigenic effects, yielding contradictory outcomes.^11^

Mesenchymal stem cells express Toll‐like receptors (TLRs) and can be used for immunomodulation by either inducing or inhibiting these receptors. When MSCs are stimulated through different TLRs, they undergo phenotypic changes, including alterations in secretion profiles, immunomodulatory capabilities, and migration abilities. The specific response and functional properties of MSCs can vary depending on the TLRs. Therefore, they are activated through them, highlighting the potential of utilizing these receptors for immunomodulation purposes.^12^ When induced via different TLRs, MSCs exhibit distinct tumorigenic properties. For instance, the MSC1 phenotype induced by the TLR4 agonist lipopolysaccharide (LPS) has been shown to reduce colony formation in ovarian, breast, and pancreatic cancer cells co‐culture models. On the other hand, the MSC2 phenotype induced by TLR3 activation has been demonstrated to increase colony formation in the same cancer cells.^13^ In contrast, when MSCs treated with LPS and their conditioned media were added to the culture medium of different cancer cell lines, including SKBR3, MDA‐MB‐231, A549, 95D, and HepG2, this increased both colony formation and proliferation of these cancer cell lines.^14^

Considering these studies, the mechanistic effects of TLR‐stimulated MSCs on tumor cells in both indirect and direct co‐culture models are not clear. Therefore, there is a need for further detailed investigations on the effects of MSCs treated with TLR4 agonists on cancer cells. Simultaneously, studies using MSCs treated with TLR4 antagonists are also required to compare their opposing effects. Understanding the role of MSCs in the tumor microenvironment will provide valuable insights for future studies related to microenvironment‐targeted therapeutic approaches. The aim of this study was to evaluate the potential therapeutic effects of TLR4 agonist and TLR4 antagonist treated ADMSCs on PDAC cells. To clarify the potential antitumorigenic effects of ADMSCs via TLR4 on Panc‐1 cells, we mechanistically assayed cell proliferation, apoptosis, colony‐forming capacity, epithelial‐mesenchymal transition (EMT), and metastasis mechanisms in both proinflammatory and anti‐inflammatory ADMSC phenotypes using TLR4 agonists and antagonists.

## 2 MATERIALS AND METHODS

### 2.1 Experiments for ADMSC isolation and optimization

#### 2.1.1 Adipose‐derived mesenchymal stem cell isolation and Immunophenotyping

In this section, the aim was to isolate and characterize the ADMSCs that will be used in the experiments. Approval was obtained from the Lokman Hekim University Non‐Interventional Ethics Committee with decision number 2021/136 (Code: 2012131). We obtained written informed consent from study participants to use liposuction materials. The discarded adipose tissue from a healthy female patient who underwent elective abdominal liposuction surgery for cosmetic purposes at Lokman Hekim Akay Hospital Plastic and Aesthetic Surgery Clinic was used. The adipose tissue obtained during the procedure was collected in a sterile tube and stored at +4°C. The adipose tissue was separated from the tumescent solution and transferred into a 10‐mL syringe, followed by centrifugation at 1500 × *g* for 8 min. The condensed adipose tissue, representing 10% of the total amount, was isolated using a sterile syringe. Collagenase enzyme (Collagenase NB 4 Standard Grade 500 mg/Cat no: S1745402 Nordmark Arzneimittel GmbH Co. KG, Uetersen, Germany) was added to the isolated condensed adipose tissue in an amount equivalent to 10%. After incubating at 37°C for 30 min, the mixture was centrifuged at 400 × *g* for 10 min to separate the stromal vascular fraction pellet from the liquid component.^15^ The final pellet obtained was resuspended in complete culture medium containing; DMEM%88, 20% FBS, 1% penicillin/streptomycin, and 1% L‐glutamine, and transferred to culture flasks (Nest Scientific USA Inc.) for further cultivation.^16^

The cultured ADMSCs were prepared according to the kit protocol (R&D Systems, Cat. No. FMC020). Specific positive antibodies (anti‐CD90+, anti‐CD105+, or anti‐CD73+), negative marker cocktail (anti‐CD45‐, anti‐CD34‐, anti‐CD11b‐, anti‐HLA‐DR, anti‐CD79‐), and 10 μL of isotype control antibody were used for the samples.^17^ The stained cell pellets were analyzed using a FACS (BD FACSAria™ III Sorter, BD Biosciences, New Jersey, USA). Fluorescence analyses were displayed as histograms using Cellquest Pro software (BD Biosciences, San Jose, CA).

#### 2.1.2 Cell culture

Adipose‐derived MSCs were grown in DMEM (Capricorn, Germany), FBS (Capricorn, Germany), 1% penicillin/streptomycin (BIOIND, Israel), and 1% L‐glutamine (Capricorn, Germany) at 37°C. Cells were used for ELISA, qRT‐PCR, and cytotoxicity experiments when they reached 70%–80% density in the culture dishes in which the experiment was performed.^13^ The ADMSCs were used in the experiments before reaching passage number three. Their morphological changes were continuously followed throughout the study.

#### 2.1.3 TLR4 agonist and antagonist treatment

The TLR4 agonist which was Ultrapure LPS from E. coli 0111:B4 (Cat no: tlrl‐3pelps), and the TLR4 antagonist which was Ultrapure lipopolysaccharide from R. Spheroids (Cat no: tlrl‐prslps), were both purchased from InvivoGen (San Diego, California) and prepared according to the manufacturer's instructions. Pro‐inflammatory and anti‐inflammatory characters were determined according to TLR4 agonist and TLR4 antagonist responses to cytokines. We defined ADMSCs stimulated with 0.5 μg/mL TLR4 agonist as proinflammatory phenotype, ADMSCs treated with 25 μg/mL TLR4 antagonist as anti‐inflammatory phenotype, and naive and untreated ADMSCs as ADMSCs only. Naïve ADMSCs were selected as the control groups.

#### 2.1.4 Cell viability assay

The MTT method was used to determine the effect of TLR4 agonists and antagonists on cell viability in ADMSCs. TLR4 agonist was applied at doses of 0, 0.01, 0.1, 1, and 10 μg/mL for 0, 24, and 48 h. For TLR4 antagonist, doses of 0.01, 0.1, 1, 10, 100, and 500 μg/mL were applied for 0, 24, and 48 h. At each time point, 10 μL of a 0.5 mg/mL MTT solution (Biotium, California, USA) was added to each well, followed by incubation for 4 h in the dark at 37°C. Then, dimethyl sulfoxide (DMSO) was added to each well to dissolve the formazan crystals. Absorbance was measured at 570 nm using a microplate reader (BioTek Synergy H1, BioTek Instruments, Winooski, VT, USA). Naïve ADMSCs were selected as the control groups.

#### 2.1.5 Cytokine secretion quantity analysis

To determine the optimal doses of TLR4 agonist and antagonist, IL‐6 was used. The TLR4 agonist was applied at doses of 0.01, 0.1, 0.5, and 1 μg/mL according to the protocol by Waterman.^18^ The TLR4 antagonist was applied at doses of 5 and 25 μg/mL for 24 h. Subsequently, the analysis of other proinflammatory cytokines was performed at a dose of 0.5 μg/mL for the TLR4 agonist and 25 μg/mL for the TLR4 antagonist. Sandwich ELISA test was conducted according to the manufacturer's protocol to measure cytokine expressions. Eight proinflammatory cytokines were analyzed: Interleukin 1‐alpha (IL‐1‐α), Interleukin 1‐beta (IL‐1β), Interleukin 6 (IL‐6), Interleukin 8 (IL‐8), Interferon‐gamma (IFN‐γ), Granulocyte macrophage colony‐stimulating factor (GM‐CSF), Monocyte chemoattractant protein‐1 (MCP‐1), and tumor necrosis factor alpha (TNF‐α) (ELISA kit, ELK Biotechnology, Wuhan). The absorbance was measured at 450 nm (BioTek Synergy H1, BioTek Instruments, Winooski, VT, USA).

#### 2.1.6 Gene expression analysis with phenotype optimization

In this experiment, we employed gene expression analysis to optimize the inflammatory phenotype. This segment focused on fine‐tuning the dosage of TLR4 agonists and TLR4 antagonists. The study evaluated the expression of various proinflammatory cytokines, namely IL‐1α, IL‐1β, IL‐6, IL‐8, TNF‐α, IFN‐γ, MCAF, and GM‐CSF, in ADMSCs. For the IL‐6 and TNF‐α gene expression analysis, the TLR4 agonist was administered at concentrations of 0.01, 0.1, 0.5, and 1 μg/mL, while the TLR4 antagonist was applied at doses of 5 and 25 μg/mL for a duration of 6 h. Subsequent to the application, TRIzol (ABP Biosciences, Wuhan, China) was utilized for RNA isolation. To evaluate gene expression, 100 ng of RNA was subjected to real‐time PCR using both the Lightcycler® 96 system (Roche Diagnostic Systems, Indianapolis, IN) and A.B.T.™ 2X qPCR SYBR‐Green Master Mix (ATLAS Biotechnology, Turkey) for qRT‐PCR analysis. The final concentration of all primers used, as listed in Table S1, was 0.1 uM. The amplification protocol involved initial steps of 55°C for 5 min and 95°C for 5 min, followed by 40 cycles of 95°C for 15 seconds, 60°C for 30 seconds, and 40°C for 1 min. A melting curve analysis ensured result specificity. To standardize the data, the threshold cycle (Ct) value of the GAPDH gene was used. Naïve ADMSCs were selected as the control groups. The experiments were conducted in triplicate, and the average Ct value was employed for subsequent calculations. The ratio of Ct values for the experimental groups was calculated accordingly, following the method outlined by Waterman et al.^18^

### 2.2 Experiments involving indirect and direct coculture

#### 2.2.1 Indirect and direct co‐culture design

In this study, the effects of both naive ADMSCs and proinflammatory/anti‐inflammatory ADMSCs on pancreatic cancer cells using direct and indirect co‐culture methods were investigated. Panc‐1 cells (ATCC CRL‐1469™) were cultured in DMEM supplemented with FBS, L‐glutamine, and penicillin/streptomycin, following the protocol outlined by Curvello et al. in 2019.^19^ In the direct co‐culture setup, ADMSCs and Panc‐1 cells were seeded into 96‐well culture plates at three different ratios: (1:1), (1:10), and (10:1). After 5 h of ADMSC seeding, a TLR4 agonist (0.5 μg/mL) and antagonist (25 μg/mL) were applied for 24 h. Following three washes with 2x phosphate buffered saline (PBS), pancreatic cancer cells were introduced, and a proliferation assay was conducted after 72 h.

For all indirect co‐culture experiments, a 10:1 ratio of ADMSCs to Panc‐1 cells was employed. Panc‐1 cells were seeded into 24‐well plates at a density of 2.5 × 10^3^ cells per well. Concurrently, ADMSCs were placed in 0.4 μm Transwells (140620, Thermo Fisher Scientific Inc, Germany) at a count of 25 × 10^3^ cells.^20^ ADMSCs in the Transwells were stimulated with the TLR4 agonist (0.5 μg/mL) and antagonist (25 μg/mL) for 24 h. After this period, ADMSCs were washed with 2x PBS and placed onto the Panc‐1 cells. Subsequent assessments included calcein staining at 48 and 96 h to measure pancreatic cell proliferation, flow cytometry analysis at 96 h to evaluate apoptosis rate and cell cycle, crystal violet staining at 96 h to assess colony formation, qRT‐PCR analysis to examine gene expression associated with metastasis and EMT mechanisms, and immunofluorescence staining at 96 h. Under co‐culture conditions, the each other effects of ADMSCs and Panc‐1 cells on the expressions of proinflammatory cytokines were analyzed by qRT‐PCR.

#### 2.2.2 Cell proliferation assay

The evaluation of the effects of naive, proinflammatory, and anti‐inflammatory characteristics of ADMSCs on the proliferation of Panc‐1 cells was conducted in both direct and indirect models. The cells were carefully washed twice with PBS and incubated with 2 μM calcein‐acetoxymethyl ester (Santa Cruz Biotechnology, Inc., Dallas, TX, USA) at 37°C for 30 min in the dark in the incubator.^13^ The live cells were viewed and photographed using a Leica DM IL fluorescent inverted microscope (Leica, Wetzlar, Germany). For each experimental group, the Panc‐1 cells in the image field were counted using the Image J software (NIH, Bethesda, Maryland, USA). The cell count and the mean values were determined for each group. Panc‐1 cells were used as the control group. Subsequently, the results were expressed as a percentage relative to the control group, by normalizing them to the control group's values.

#### 2.2.3 Cell cycle analysis

Cell cycle analysis aimed to determine the cell cycle phase based on the DNA content of cells.^21^ For this study, the Cell Cycle Analysis Kit (THOR‐CCK‐100, Thorvacs Biotechnology, Turkey) was used following the manufacturer's protocol. The analysis was performed at 96 h in the indirect co‐culture. After preparing the cells according to the protocol, propidium iodide was added and incubated at 37°C for 30 min. After reading with the NovoCyte Flow Cytometer, histograms were generated using NovoExpress Software (1.6.1). Panc‐1 cells were used as the control group.

#### 2.2.4 Apoptosis analysis

A nucleic acid dye capable of distinguishing live and necrotic cells was used for apoptosis analysis.^22^ For this study, the Fluorescein isothiocyanate (FITC) Annexin V Apoptosis Detection Kit with 7‐Aminoactinomycin D (7‐AAD) [Tonbo Bioscience, Cat. No. 35‐6410 (San Diego, United States)] was utilized following the protocol provided with the kit. This analysis was performed as described in the indirect co‐culture model. At the 96th hour of the experiments, the co‐cultures were terminated, and the cells in the 24‐well culture plates were removed by trypsinization and transferred to eppendorf tubes. The cells were then centrifuged at 1000 × *g* for 5 min, and 10,000 cells were separated for analysis. Annexin V binding buffer, provided in the kit at a concentration of 1x, was added to each cell pellet in a volume of 500 μL per sample. Subsequently, 100 μL of binding buffer, 5 μL of FITC Annexin V stain, and 5 μL of 7‐ AAD were added to the pellet for each sample. After incubation for 15 min at 25°C in the dark, readings were taken using the NovoCyte Flow Cytometer, and graphs were generated using NovoExpress Software (1.6.1). Panc‐1 cells were used as the control group.

#### 2.2.5 Gene expression analysis of Panc‐1 cells

We aimed to determine the effect of three different inflammatory phenotypes of ADMSCs on the expression levels of EMT and metastasis‐related genes in Panc‐1 cells. After 96 h, following RNA isolation, the expression analysis of several genes including Glyceraldehyde‐3‐Phosphate Dehydrogenase (GAPDH), Cyclin D1 (CCND1), E‐cadherin (CDH1), Vimentin (VIM), Zinc finger E‐box binding homeobox 1 (ZEB1), as well as Matrix Metalloproteinase 2 (MMP2), Kinase Insert Domain Receptor (KDR), Claudin 1 (CLDN1), Matrix Metalloproteinase 9 (MMP9), Tissue Inhibitor of Metalloproteinase 1 (TIMP1), Insulin‐like Growth Factor 2 (IGF2R), and Alpha‐1 Type I Collagen (COL1A1) was performed using qRT‐PCR with specific primers. Panc‐1 cells were used as the control group. All primers used are listed in Table S1. PCR and gene expression analysis were carried out under the same conditions as described in the previous section.

#### 2.2.6 Immunofluorescent (IF) and Immunohistochemistry (IHC) staining

This analysis was conducted to visualize the effect of ADMSCs on the proteins controlling the EMT mechanism in Panc‐1 cells. For immunofluorescence and Immunohistochemistry (IHC) staining, Panc‐1 cells were cultured on 13 mm uncoated glass coverslips in an indirect co‐culture model for 96 h. After removing the medium, cells were incubated in 4% Paraformaldehyde (PFA) (SERVA, Germany), 0.1% Triton X‐100, and 1% Bovine serum albumin (BSA) solutions. Then, they were incubated with E‐cadherin antibodies (E‐AB‐31261, Elabscience, Texas, USA) at a 1:100 dilution in one group. In another group, cells were incubated with Vimentin antibodies (E‐AB‐67478, Elabscience, Texas, USA) at a 1:200 dilution. For IF staning; the cells were incubated with Goat Anti‐Rabbit IgG H&L Alexa Fluor® 488 secondary antibodies (Abcam, Alexa Fluor® 488, ab150077, Cambridge, United Kingdom) at a 1:200 concentration in the dark for 30 min. Then, they were incubated with 4′,6‐diamidino‐2‐phenylindole (DAPI) (SERVA, Germany) dye at a concentration of 0.1 μg/mL. The images of the stained samples were captured using a Leica DM IL fluorescent microscope. For IHC staining; 2‐step plus Poly‐HRP Anti Rabbit IgG Detection System with DAB Solution (Cat no: E‐IR‐R215) was used according to the protocol. Then, they were incubated with Hematoxylin (SERVA, Germany) dye. The images of the stained samples were captured using a Leica DM IL light microscope.

#### 2.2.7 Colony forming unit (CFU) assay

This analysis was conducted to determine the effects of naive, proinflammatory, and anti‐inflammatory phenotype ADMSCs on the colony formation capacity of Panc‐1 cells. Panc‐1 cells were seeded in six‐well culture plates at a density of 4000 cells per well in triplicate. Subsequently, pure, proinflammatory, and anti‐inflammatory phenotype ADMSCs were added and cultured at 37°C for 14 days. After incubation, the cells were fixed, stained with 0.5% (w/v) crystal violet solution, and the absorbance was measured at 595 nm using a microplate reader (Biotek Synergy H1, Biotek Instruments, Winooski, VT, USA). Images were captured using an inverted microscope (Leica DM IL, Leica, Wetzlar, Germany). The schematic representation of all experiments described in the material method section is given in Figure S1.

### 2.3 Statistical analysis

GraphPad Prism 8.1 software (GraphPad, San Diego, CA, USA) was used for the graphical representation and statistical analysis of the results provided as mean and standard deviation (SD). Nonparametric one‐way or two‐way ANOVA tests were performed for all statistical analyses. Subsequently, Tukey's and Dunnett post hoc tests were applied for adjustments. ADMSCs and Panc‐1 cells without any treatment were selected as the control groups. A significance level of *p* < 0.05 was considered statistically significant and denoted by an asterisk (*). The significance level was further detailed in the analyses using the following symbols: (*p < 0.05, **p < 0.01, ***p < 0.001, ****p < 0.0001).

## 3 RESULTS

### 3.1 Cell viability in ADMSCs modulated by TLR4‐mediated signaling

The isolated cells were morphologically identified as ADMSCs, displaying a characteristic spindle‐shaped morphology (Figure S2A). Flow cytometry analysis of the ADMSCs, as depicted in Figure S2B, revealed distribution graphs based on staining. Specifically, 91.9% of the stained cells expressed CD73, 89.9% expressed CD90, and 90.1% expressed CD105, validating their mesenchymal stem cell identity. Moreover, the proportion of cells negative for the marker cocktail was determined to be 5.06%, further supporting the confirmation of cultured cells as mesenchymal stem cells based on surface markers (Figure S2B).

**FIGURE 1:**
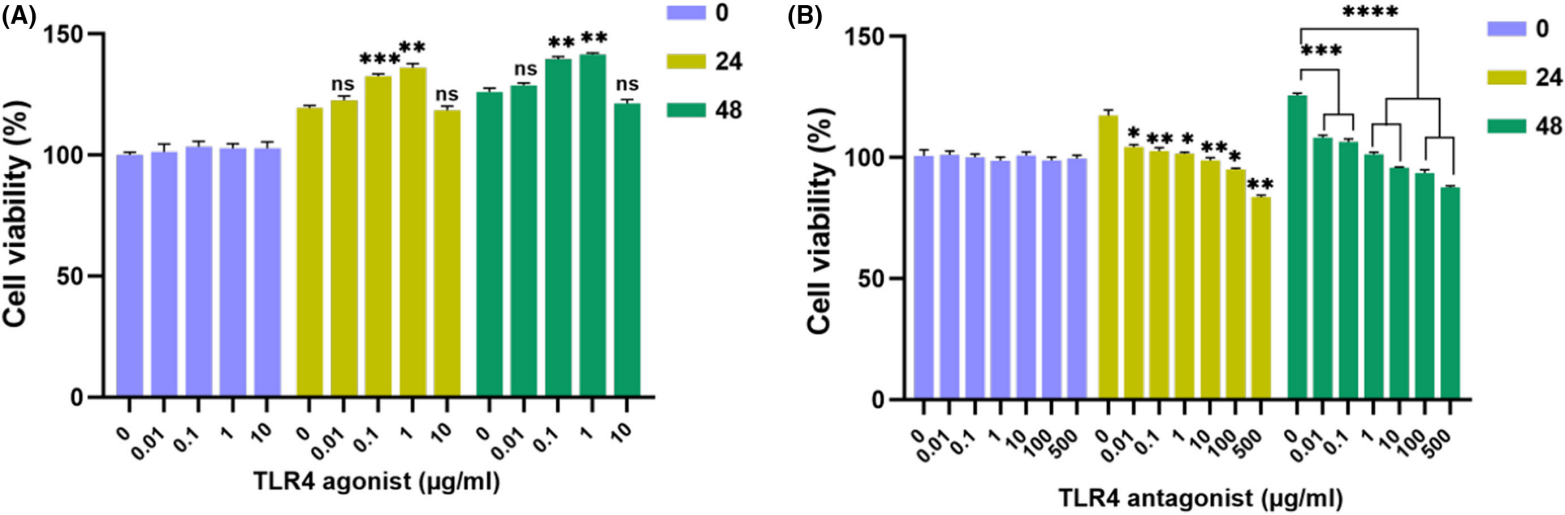
Represents the effect of TLR4 agonist and TLR4 antagonist different doses viability of ADMSCs. The cells were treated to various doses of TLR4 agonist (A) (0.01, 0.1, 1, and 10 μg/mL)and TLR4 antagonist (B) (0.01, 0.1, 1, 10, 100, and 500 μg/mL) for 48 h to to decide the dose to be used in future experiments. The results are presented as the mean of relative viability from three independent experiments (mean ± SD, *n* = 3). Statistical significance is represented by stars: * for *p* < 0.05, ** for *p* < 0.01, *** for *p* < 0.001, and **** for *p* < 0.0001, ns:not significant indicating a significant difference from the control group. The data were analyzed by two‐way ANOVA followed by Tukey post hoc testing.

Viability assessments of cells were conducted to ascertain the potential cytotoxic effects of TLR4 agonist and antagonist on ADMSCs. The TLR4 agonist was administered at varying doses (0.01, 0.1, 1, and 10 μg/mL) over different time periods (0, 24, and 48 h). After 24, the control group exhibited a 19.4% increase in viability. Meanwhile, the 0.01, 0.1, 1 and 10 μg/mL groups expressed an increase as 22.5%, 32.6%, 36.5% and 18.43%, respectively, compared to the control group. Following 48, the control group's viability rose by 26%, the 0.01 μg/mL group showed a 28.7% increase, the 0.1 μg/mL group exhibited a 39.7% increase, the 1 μg/mL group demonstrated a 41.5% increase, and the 10 μg/mL group had a 21.3% increase (Figure 1A). Notably, the activation mediated by TLR4 was observed to impact cell viability. Furthermore, the TLR4 antagonist was applied at varying doses (0.01, 0.1, 1, 10, 100, and 500 μg/mL) for different time intervals (0, 24, and 48). After 24 h, the control group displayed a 17.3% increase in viability. Meanwhile, the 0.01 μg/mL group showed a 4.3% increase, the 0.1 μg/mL group exhibited a 2.6% increase, and the 1 μg/mL group demonstrated a 1.6% increase. Notably, the 10 μg/mL group indicated a 1.3% decrease, the 100 μg/mL group exhibited a 4.9% decrease, and the 500 μg/mL group displayed a 16.3% decrease. Following 48 h, the control group showed a 25.6% increase in viability, the 0.01 μg/mL group displayed an 8.2% increase, the 0.1 μg/mL group demonstrated a 6.3% increase, and the 1 μg/mL group had a 1.2% increase. The 10 μg/mL group indicated a 4.3% decrease, the 100 μg/mL group showed a 6.5% decrease, and the 500 μg/mL group displayed a 13.4% decrease (Figure 1B).

### 3.2 TLR4 activation stimulates upregulation and secretion of proinflammatory cytokines in ADMSCs

**FIGURE 2:**
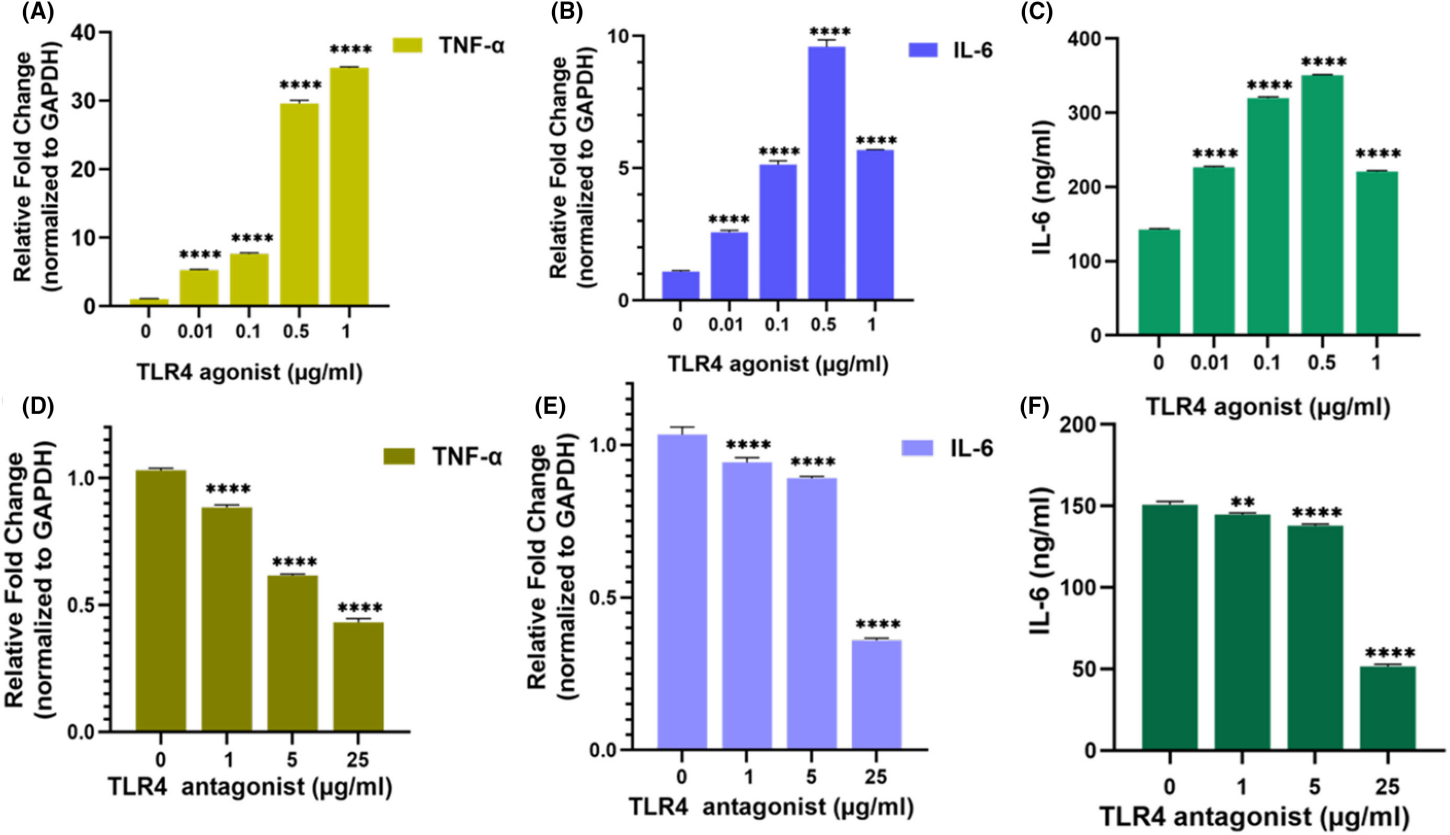
Indicates the effect of TLR4 agonist (0.01, 0.1, 0.5, and 1 μg/mL) and TLR4 antagonist (1, 5 and 25 μg/mL) on the expression of IL‐6, TNF‐α genes and IL‐6 protein level protein in the supernatant. The effects of TLR4 mediated signaling activation and inhibition on proinflammatory cytokines gene expression are presented. The TLR4 agonist dose‐dependently induced the expression of TNF‐α (A) and IL‐6 (B) genes. TLR4 antagonist, on the other hand, dose‐dependently inhibited the expression of IL‐6 (E) and TNF‐α (D) genes. TLR4 antagonist increases the secretion level of IL‐6 cytokine (C) to the supernatant while TLR4 antagonist decreases the level of IL‐6 (F). The untreated ADMSCs served as the control. The results were normalized to the expression of GAPDH. Statistical significance is represented by stars: ** for *p* < 0.01, and **** for *p* < 0.0001, indicating a significant difference from the control group. The data were analyzed by two‐way ANOVA followed by Tukey post hoc testing.

The experiments in this section were conducted to determine the effects of TLR4 agonist and antagonist doses on ADMSCs at the mRNA and protein levels, with the aim of utilizing them to induce a proinflammatory or anti‐inflammatory phenotype through stimulation and inhibition via TLR4. When the gene expression in the control group, where no TLR4 agonist was applied, was considered as a value of 1, the TNF‐α gene showed an increase of 5.2‐fold at 0.01 μg/mL dose, 7.6‐fold at 0.1 μg/mL dose, 29.5‐fold at 0.5 μg/mL dose, and 34.8‐fold at 1 μg/mL dose (Figure 2A). The IL‐6 gene exhibited a 2.5‐fold increase at 0.01 μg/mL dose, 5.1‐fold increase at 0.1 μg/mL dose, 9.5‐fold increase at 0.5 μg/mL dose, and 5.6‐fold increase at 1 μg/mL dose (Figure 2B). The IL‐6 protein in the supernatant was measured as 142.47 ng/mL in the control group, 226.67 ng/mL at 0.01 μg/mL dose, 319.77 ng/mL at 0.1 μg/mL dose, 350.58 ng/mL at 0.5 μg/mL dose, and 220.94 ng/mL at 1 μg/mL dose (Figure 2C). When the gene expression in the control group, where no TLR4 antagonist was applied, was considered as a value of 1, the TNF‐α gene exhibited a decrease of 0.88‐fold at 1 μg/mL dose, 0.61‐fold at 5 μg/mL dose, and 0.43‐fold at 25 μg/mL dose (Figure 2D). The IL‐6 gene showed a decrease of 0.94‐fold at 1 μg/mL dose, 0.89‐fold at 5 μg/mL dose, and 0.35‐fold at 25 μg/mL dose (Figure 2E). The IL‐6 protein in the supernatant was measured as 150.7 ng/mL in the control group, 144.7 ng/mL at 1 μg/mL dose, 137.9 ng/mL at 5 μg/mL dose, and 51.6 ng/mL at 25 μg/mL dose (Figure 2F). Based on the highest IL‐6 value, the decision was made to use a TLR4 agonist dose of 0.5 μg/mL in further experiments. According to the lowest IL‐6 value, 25 μg/mL dose of TLR4 antagonist was chosen for our further experiments. After determining the application doses, the gene expression changes of other proinflammatory cytokines (IL‐1‐α, IL‐1β, IL‐8, IFN‐γ, GM‐CSF, MCAF, TNF‐α) (Table 1) and cytokine secretion levels (Table 2) were provided. Following the presentation of the levels of these cytokines, the group that received a dose of 0.5 μg/mL TLR4 agonist was referred to as the proinflammatory MSC group, while the group that received a dose of 25 μg/mL TLR4 antagonist was referred to as the anti‐inflammatory MSC group. As a result of the optimizations in this section, the proinflammatory and anti‐inflammatory characteristics of ADMSCs were utilized in direct and indirect co‐cultures.

**TABLE 1:** TLR4 agonist, 0.5 μg/mL, and TLR4 antagonist, 25 μg/mL, mediated the changes in gene expression levels of proinflammatory cytokines in ADMSCs.

Gene name	TLR4 agonist 0.5 μg/mL (Fold Change)	TLR4 antagonist 25 μg/mL (Fold Change)
IL‐1α	6.92 ± 0.345	0.073 ± 0.007
IL‐1β	4.25 ± 0.061	0.23 ± 0.041
IL‐8	21.42 ± 1.244	0.473 ± 0.034
GM‐CSF	2.17 ± 0.082	0.122 ± 0.010
MCAF	2.38 ± 0.311	0.549 ± 0.043
IFN‐γ	1.18 ± 0.096	0.955 ± 0.036

**TABLE 2:** The supernatant protein levels of proinflammatory cytokines at TLR4 agonist dose of 0.5 μg/mL and TLR4 antagonist dose of 25 μg/mL in ADMSCs.

Protein name (pg/mL)	0 (pg/mL)	TLR4 agonist 0.5 μg/mL (pg/mL)	TLR4 antagonist 25 μg/mL (pg/mL)
IL‐1α	340.71 ± 10.12	603.7 ± 5.527	180.6 ± 9.95
IL‐1β	232.98 ± 7.48	308.2 ± 2.18	155.77 ± 2.93
IL‐8	65116.66 ± 1444.24	749366.66 ± 3000.55	4446.66 ± 45.092
GM‐CSF	588.66 ± 4.72	9867.27 ± 35.02	464.54 ± 4.83
MCP‐1	225.51 ± 5.894	2.38 ± 0.311	0.549 ± 0.043
IFN‐γ	52.613 ± 0.655	2757.51 ± 40.379	117.095 ± 5.260
TNF‐α	467.427 ± 7.75	5333.684 ± 76.782	315.181 ± 5.011

### 3.3 Increased presence of both naive and proinflammatory ADMSCs leads to the suppression of Panc‐1 cell proliferation in direct co‐culture

**FIGURE 3:**
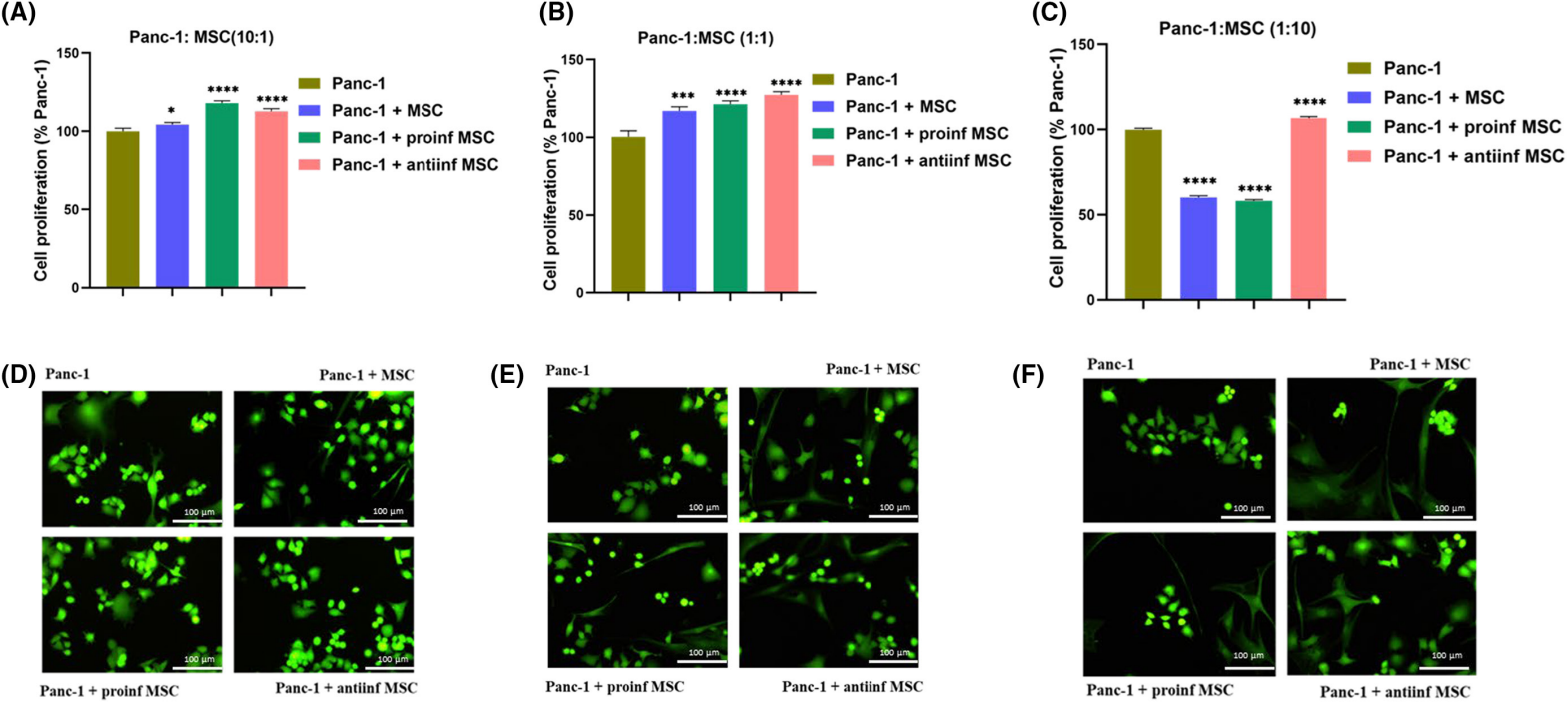
Depicts the results of treatingnaive, proinflammatory and antiinflammatory ADMSCs with Panc‐1 direct coculture different rate for 72 h. Graph A–C presents the quantified cell proliferation rate. Panel (D–F) shows representative images of cell proliferation assessed using Calcein AM staining at 20x magnification. Proliferation results of the group with a (10) Panc‐1: ADMSC (1) ratio of at the end of 72 h (A) and image (D) are shown. Proliferation results of the group with a (1) Panc‐1: ADMSC (1) ratio of at the end of 72 h (B) and image (E) are shown. Proliferation results of the group with a (1) Panc‐1: ADMSC (10) ratio of at the end of 72 h (C) and image (F) are shown. The untreated Panc‐1 cells served as the control. Data are given with mean ± SDn = 3). Statistical significance indicated by stars, where *p < 0.05, ***p < 0.001, and ****p < 0.0001. Only results that were significant compared to the control group are shown. The data were analyzed by one‐way ANOVA followed by Dunnet post hoc testing.

In this investigation, we examined the influence of co‐culturing Panc‐1 cells with ADMSCs exhibiting their native, proinflammatory, and anti‐inflammatory phenotypes. The co‐cultures were established at ratios of 10:1, 1:1, and 1:10 for Panc‐1 cells and ADMSCs. Calcein staining‐based cell counting was performed exclusively at the 72 mark. For the 10:1 Panc‐1:ADMSC ratio group, at the end of the 72‐h period, proliferation rates were determined as follows: 104.2% in the Panc‐1 + ADMSC group, 117.9% in the Panc‐1 + proinflammatory ADMSC group, and 112.8% in the Panc‐1 + anti‐inflammatory ADMSC group (Figure 3A,D). In the 1:1 ratio group, proliferation rates were observed as 117% in the Panc‐1 + ADMSC group, 121.3% in the Panc‐1 + proinflammatory ADMSC group, and 127.3% in the Panc‐1 + anti‐inflammatory ADMSC group. Notably, significant distinctions were only observed between Panc‐1 and all experimental groups, with corresponding significance levels indicated (Figure 3B,E) (*p* < 0.05). Within the 1:10 ratio group, the 72‐h proliferation rates were 60.1% in the Panc‐1 + ADMSC group, 58.2% in the Panc‐1 + proinflammatory ADMSC group, and 106.8% in the Panc‐1 + anti‐inflammatory ADMSC group (Figure 3C,F). Once again, significant differences were evident solely between Panc‐1 and the experimental groups, with associated significance levels denoted (Figure 3C,F) (*p* < 0.05). Considering the proliferation analysis outcomes and the notable disparities observed, the 1:10 Panc‐1:ADMSC ratio was selected for subsequent experiments, aligning with cytotoxicity considerations.

### 3.4 The proliferation of Panc‐1 cells is suppressed as a result of S phase arrest induced by both naive and proinflammatory ADMSC phenotypes

**FIGURE 4:**
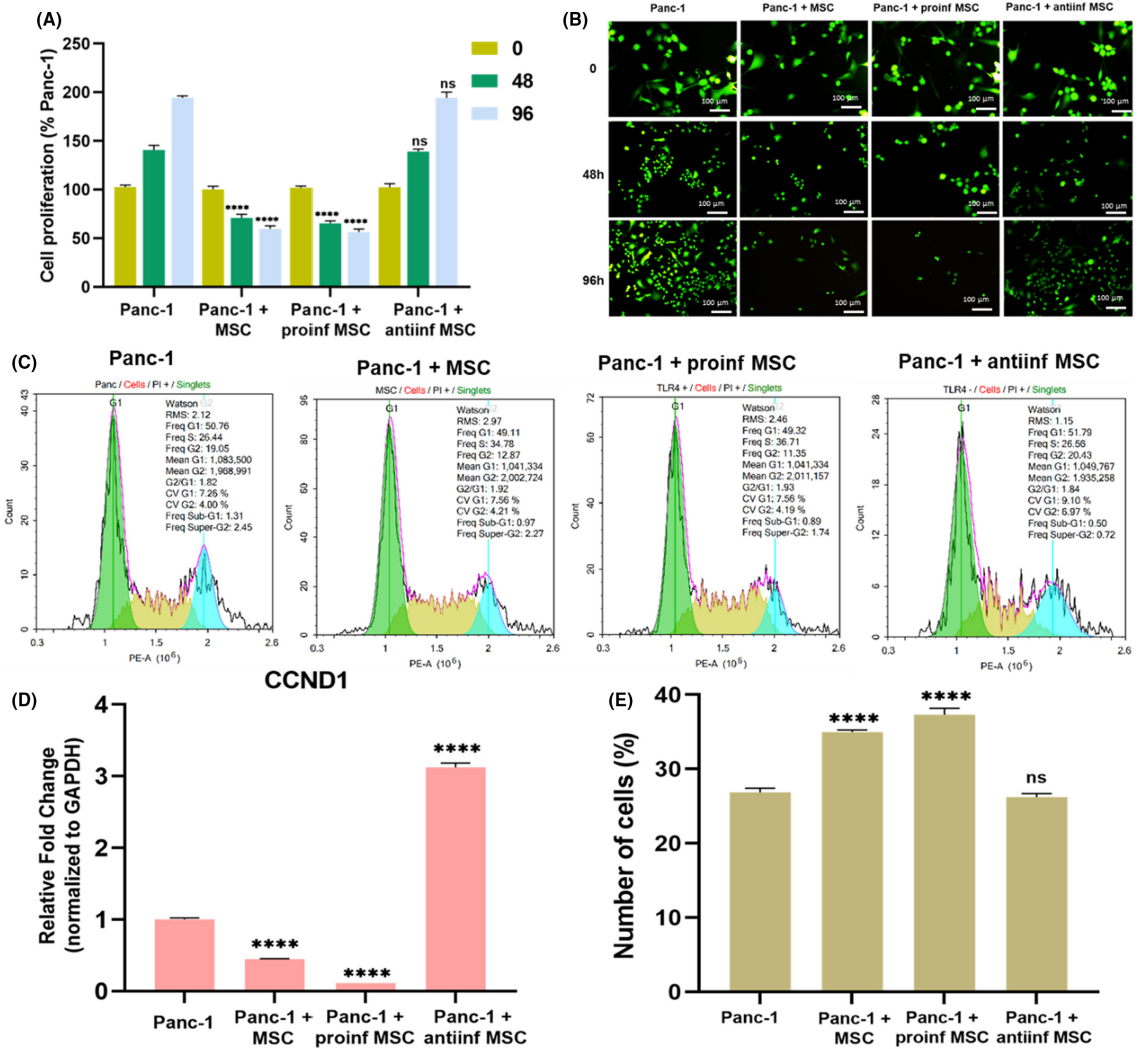
Shows the effect of indirect culturing of Panc‐1 cells with naive ADMSCs, pro‐inflammatory ADMSCs and anti‐inflammatory ADMSCs at a ratio of 1:10 on the proliferation and cell cycle of Panc‐1 cells. Proliferation results of 48 h and 96 h in three different coculture experimental groups and control groups (Panc‐1) were given as percentage graphic (A) and Calcein staining total images (B). Cell cycle histograms of the experimental groups (C) and the number of cells belonging to the S phase are given graphically (E). The expression levels of the CCDN1 gene belonging to the experimental groups were graphed (d). The untreated cells served as the control. The results were normalized to the expression of GAPDH. Statistical significance is represented by stars: **** for *p* < 0.0001, indicating a significant difference from the control group. The data were analyzed by two‐way ANOVA followed by Tukey post hoc testing.

This analysis was performed to determine the effect of co‐culturing Panc‐1 cells withnaive ADMSCs, proinflammatory ADMSCs, and anti‐inflammatory ADMSCs on the proliferation of Panc‐1 cells at a 1:10 ratio at 48 and 96 h. At the end of the 96 h, while the Panc‐1 group showed a 94.3% increase, the Panc‐1 + MSC group showed a 41.4% decrease, the Panc‐1+ proinf MSC group showed a 43.7% decrease, and the Panc‐1 + antiinf MSC group showed a 94.3% increase (Figure 4A). A statistically significant difference was observed between the Panc‐1, Panc‐1 + MSC, and Panc‐1 + proinf MSC groups at both 48 and 96 h (*p* < 0.0001). However, there was no significant difference between the Panc‐1 group and the Panc‐1 + antiinf MSC group at both time points (p > 0.05). These results indicate an antiproliferative effect of both MSCs and proinflammatory MSCs at this ratio.

At the end of the 96th hour, the percentages of cells in the G1, S, and G2 phases were as follows: in the Panc‐1 group, 51.05%, 26.8%, and 18.7%; in the Panc‐1 + MSC group, 48.6%, 34.9%, and 13.1%; in the Panc‐1 + proinf MSC group, 48.7%, 37.3%, and 11.6%; and in the Panc‐1 + antiinf MSC group, 51.3%, 26.2%, and 20.6% (Figure 4C). Significant differences were observed in the G1 and S phases between Panc‐1 and Panc‐1 + MSC, Panc‐1 + proinf MSC groups (*p* < 0.05). In the G2 phase, significant differences were observed between Panc‐1 and all experimental groups (*p* < 0.0001). When the expression of the CCND1 gene, also known as Cyclin D1, was considered as 1 in the Panc‐1 group, it was found to be 0.449 in the Panc‐1 + MSC group, 0.115 in the Panc‐1 + proinf MSC group, and 3.121 in the Panc‐1 + antiinf MSC group (Figure 4D). The analysis revealed that MSCs and proinflammatory MSCs increased the percentage of cells in the S phase (Figure 4E) and confirmed the antiproliferative effect.

### 3.5 The naive and proinflammatory ADMSC phenotypes induce apoptotic stimulation of Panc‐1 cells through the TNF‐α and IFN‐γ signaling pathways

**FIGURE 5:**
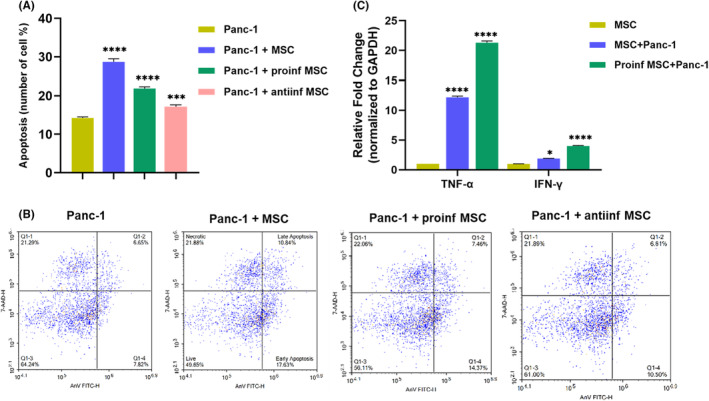
Exhibits the effect of indirect coculturing of Panc‐1 cells with naive ADMSCs, pro‐inflammatory ADMSCs and anti‐inflammatory ADMSCs at a ratio of 1:10 on the apoptosis of Panc‐1 cells. Percentage graph (A) and plot graphs (B) of the sum of 96‐h early and late apoptosis results in three different coculture experimental groups and control groups (Panc‐1). Expressions of TNF‐α and IFN‐γ genes of Panc‐1 cells cocultured with naive ADMSCs and proinflammatory ADMSCs are given (C). The untreated cells served as the control. The results were normalized to the expression of GAPDH. Statistical significance is represented by stars: * for *p* < 0.05, *** for *p* < 0.001, and **** for *p* < 0.0001, indicating a significant difference from the control group. The data were analyzed by one‐way ANOVA followed by Dunnet post hoc testing.

One of the best characterized ligands of the TNF receptor family is known as TNF‐α. TNF‐α secreted from ADMSC induces apoptosis in Panc‐1 cells.^23^ IFN‐γ acts as a cytotoxic cytokine together with granzyme B and perforin to initiate apoptosis in tumor cells. Therefore, increased expression of IFN‐γ in ADMSCs cells affects apoptosis of Panc‐1 cells.^24^ This analysis was conducted to determine the effect of co‐culturing Panc‐1 cells with unstimulated MSCs, proinflammatory MSCs, and anti‐inflammatory MSCs at a ratio of 1:10 at 96 h, aiming to investigate their impact on the apoptosis of Panc‐1 cells. At the end of the 96th hour, the total cell percentages in early and late apoptosis were as follows: in the Panc‐1 group, 14.2%; in the Panc‐1 + MSC group, 28.6%; in the Panc‐1 + proinf MSC group, 21.8%; and in the Panc‐1 + antiinf MSC group, 17.1% (Figure 5A). Significant differences were found between all experimental groups when compared with the Panc‐1 group, and their significance levels were indicated (*p* < 0.05). Distribution graphs generated as a result of flow cytometry analysis were provided (Figure 5B). Based on this analysis, it was evident that ADMSCs possess inherent proapoptotic characteristics; however, the activation of TLR4 did not augment this property. According to the gene expression analysis conducted to elucidate the mediators responsible for the apoptotic effect, TNF‐α expression was found to be 12.2 in the Panc‐1 + MSC group and 21.3 in the Panc‐1 + proinf MSC group. IFN‐γ expression was found to be 1.8 in the Panc‐1 + MSC group and 4.9 in the Panc‐1 + proinf MSC group (Figure 5C).

### 3.6 The naive and proinflammatory phenotypes of ADMSCs inhibit EMT, colony forming capacity and metastasis related gene expression in Panc‐1 cells, while promoting the anti‐inflammatory phenotype

**FIGURE 6:**
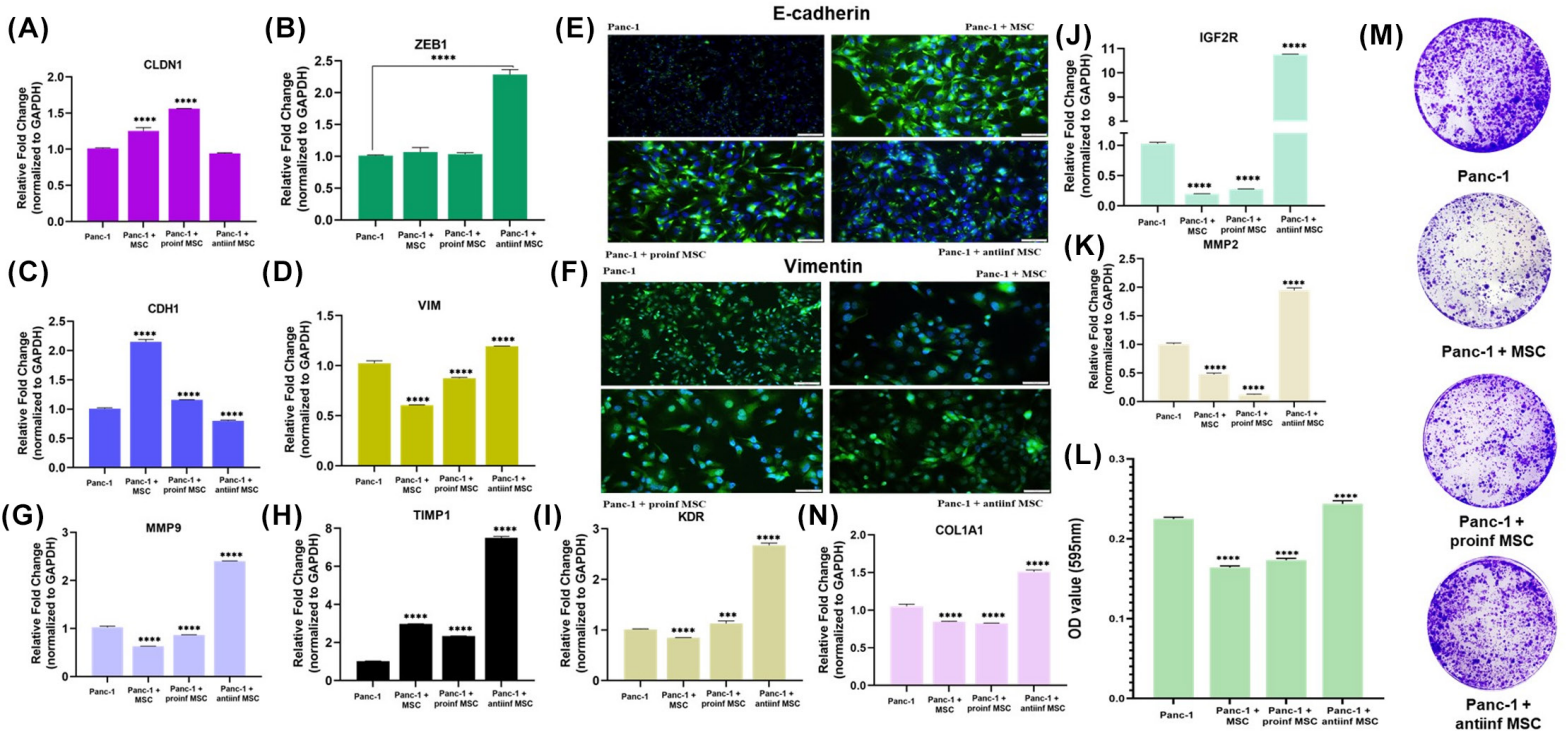
Demonstrate the 96 h effect of indirect co‐culture of Panc‐1 cells with naive ADMSCs, pro‐inflammatory ADMSCs and anti‐inflammatory ADMSCs at a ratio of 1:10 on the metastasis, EMT mechanism and colony formation capacity of Panc‐1 cells. Fold change graphs of CLDN1(A), ZEB1(B), CDH1(C) and VIM(D) gene expression of genes related to EMT mechanism are given. Immunofluorescent staining pictures of E‐cadherin (E) and Vimentin (F) proteins were given. Gene expression fold change graphs of metastasis‐related genes belonging to the experimental groups MMP9 (G), TIMP1 (H), KDR (I), COL1A1 (N), IGF2R (J) and MMP2 (K) were given. Colony forming units (CFU) test of the experimental groups OD result graph (L) and colony pictures (M) are given. The untreated cells served as the control. The results were normalized to the expression of GAPDH. Statistical significance is represented by stars: *** for *p* < 0.001, and **** for *p* < 0.0001, indicating a significant difference from the control group. The data were analyzed by one‐way ANOVA followed by Dunnet post hoc testing.

This analysis was conducted at 1:10 ratio solely at 96 h, aiming to determine the effect of naive MSCs, proinflammatory MSCs, and anti‐inflammatory MSCs the genes associated with cancer‐related mechanisms in Panc‐1 cells. The GAPDH gene was used as a control gene to determine relative gene expression. The expressions of CDH1, VIM, ZEB1, MMP2, KDR, CLDN1, MMP9, TIMP1, IGF2R, and COL1A1 genes were analyzed among the four experimental groups. The relative fold change was calculated considering the Panc‐1 group as 1, and the graphs in Figure 6A–K were provided along with statistical analyses. Significant differences were found between all experimental groups and the Panc‐1 control group in CCND1, CDH1, VIM, MMP9, TIMP1, and IGF2R genes (*p* < 0.0001). In the ZEB1 gene, a significant difference was found only between Panc‐1 and Panc‐1 + antiinf MSC groups (*p* < 0.0001). Significant differences were also found in the KDR gene between Panc‐1 and other groups. In the CLDN1 gene, a significant difference was found between Panc‐1 and Panc‐1 + MSC and Panc‐1 + proinf MSC groups (*p* < 0.0001). Based on these analyses, an increase in the expression of tumor suppressor genes was observed in the naive MSC and proinflammatory MSC groups, while a decrease was observed in oncogenes.

This analysis was conducted at a 1:10 ratio solely at 96 h, aiming to determine the effect of co‐culturing Panc‐1 cells with naive MSCs, proinflammatory MSCs, and anti‐inflammatory MSCs on the E‐cadherin and Vimentin proteins, which are associated with the epithelial‐mesenchymal transition (EMT) mechanism in Panc‐1 cells. Immunostaining of E‐cadherin (Figure 6E) and Vimentin (Figure 6F) proteins, along with DAPI nuclear staining, was performed, and the images were provided in Figure 6. Immunohistochemistry staining pictures of E‐cadherin and Vimentin proteins were given (Figure S3). It was observed that the expression of E‐cadherin protein was lower in the Panc‐1 and Panc‐1 + antiinf MSC groups compared with the Panc‐1 + MSC and Panc‐1 + proinf MSC groups. On the other hand, the expression of Vimentin protein was higher in the Panc‐1 and Panc‐1 + antiinf MSC groups compared to the Panc‐1 + MSC and Panc‐1 + proinf MSC groups. From these observations, it can be concluded that MSCs, especially those with a proinflammatory character, inhibit the EMT process in Panc‐1 cells. The OD results for evaluating colony formation were as follows: the average Optical density (OD) value for the Panc‐1 group was 0.225, for the Panc‐1 + MSC group was 0.164, for the Panc‐1 + proinf MSC group was 0.173, and for the Panc‐1 + antiinf MSC group was 0.244 (Figure 6L). Images of the colonies were shown in Figure 6M.

### 3.7 In a shared environment, ADMSCs and Panc‐1 cells mutually interact to regulate each other's proinflammatory immune responses

**FIGURE 7:**
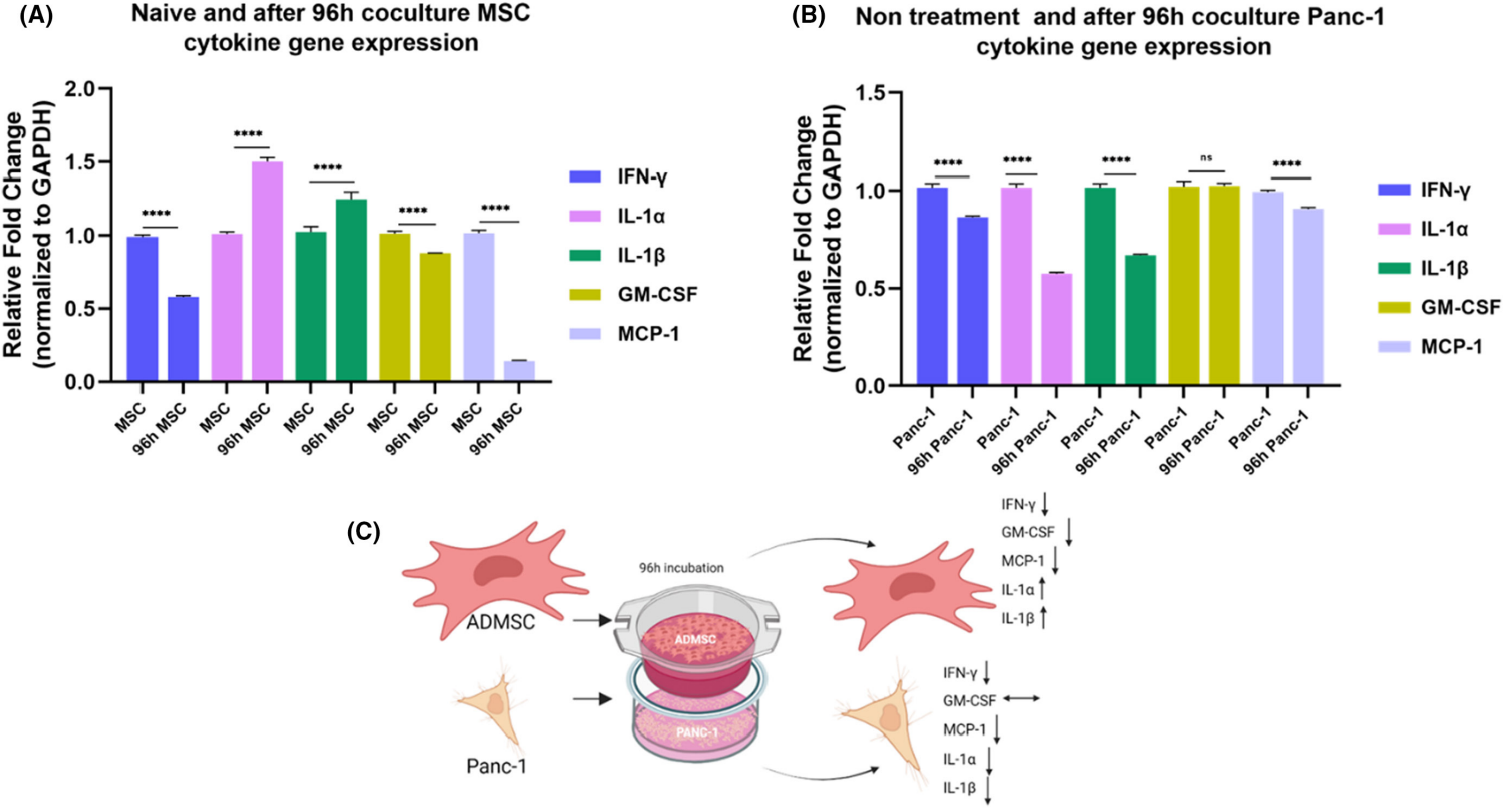
Display gene expression plots of proinflammatory cytokines of ADMSC and Panc‐1 cells indirectly cocultured for 96 h. The gene expression fold changes of IFN‐γ, IL‐1α, IL‐1β, GM‐CSF and MCP‐1 genes were given as MSC(A) and Panc‐1 (B). The figure summarizing these cytokine expressions after 96 h is given (C). The untreated cells served as the control. The results were normalized to the expression of GAPDH. Statistical significance is represented by stars: **** for *p* < 0.0001, indicating a significant difference from the control group. The data were analyzed by one‐way ANOVA followed by Dunnet post hoc testing.

This analysis was conducted at a 1:10 ratio solely at 96 h, aiming to determine the effect of co‐culturing Panc‐1 cells with unstimulated MSCs on the changes in the gene expression levels of proinflammatory cytokines. After 96 h of co‐culture, the fold changes in gene expression in ADMSCs were as follows: IFN‐γ: 0.58, IL‐1α: 1.50, IL‐1β: 1.24, GM‐CSF: 0.87, MCP‐1: 0.14 (Figure 7A). In Panc‐1 cells, the fold changes were as follows: IFN‐γ: 0.85, IL‐1α: 0.57, IL‐1β: 0.66, GM‐CSF: 1.01, MCP‐1: 0.90 (Figure 7B). Based on these analyses, it can be concluded that the cells influence each other as part of an immune response (Figure 7C).

## 4 DISCUSSION

In this study, we examined the effects of both naive MSCs and proinflammatory and anti‐inflammatory MSC phenotypes induced by stimulation and inhibition via TLR4 on Panc‐1 cells. In direct coculture, proliferation analysis at ratios of 10:1, 1:1 and 1:10 showed that (Panc‐1) decreased cell proliferation at a ratio of 1:10 (MSC). In indirect coculture, both naive MSCs and proinflammatory MSCs at a ratio of 1:10 decreased the proliferation and colony forming capacity of Panc‐1 cells and increased the apoptosis rate and the duration of S phase. Expression of EMT mechanism (CDH1, VIM, ZEB1, CLDN1), metastasis (MMP2, KDR, PLAU, MMP9, TIMP1, COL1A1), proliferation‐related genes (CCND1, IGF2R) and proinflammatory cytokines were found to be regulated during coculture.

In our study, using the same protocol, the expression of the IL‐6 gene increased 2.5‐fold with a 10 ng/mL dose after 6 h of application. The protein level was 142.47 ng/mL in the control group and 226.67 ng/mL with a dose of 0.01 μg/mL. The closest value to 350 and 400 ng/mL was reached with a dose of 0.5 μg/mL. Waterman et al. demonstrated that MSCs release immunosuppressive factors through TLR3 activation, while they secrete more proinflammatory factors through TLR4 activation upon LPS stimulation.^18^ MSCs stimulated via TLR4 are referred to as MSC1, and their specific markers are IL‐6 and IL‐8. MSC1 is suggested to reduce tumor growth.^25^ In order to induce the MSC1 phenotype, Waterman et al. treated the cells with 10 ng/mL LPS for more than 4 h for gene‐level responses, and for 1 h with a dose of 10 ng/mL LPS for protein‐level responses, followed by incubation with LPS‐free medium for 48 h. The amount of IL‐6 increased 3‐fold at the gene level and reached 400 ng/mL at the protein level compared to the control group.^18^ This may be attributed to the different TLR4 agonist used and the different origin of the MSCs. In the same study, a cDNA array analysis of MSCs in the MSC1 phenotype showed a 42‐fold increase in TNF‐α expression. In our study, the TNF‐α gene showed a 5.2‐fold increase with a dose of 0.01 μg/mL, a 29.5‐fold increase with a dose of 0.5 μg/mL, and a 34.8‐fold increase with a dose of 1 μg/mL. These data indicate the activation of TLR4 signaling. When MSCs were treated with a different TLR4 agonist, LPS, at doses of 0, 10, 100, and 1000 ng/mL, it was observed that especially at doses of 100 and 1000 ng/mL, MSC proliferation was highly induced for 6 days.^26^ In both studies, it was observed that TLR4 agonists increased cell viability and proliferation without causing cytotoxic effects.

The TLR4 antagonist we used, LPS‐RS, has previously been shown to significantly reduce the expression of IL‐6 and TNF‐α genes when co‐administered with 1 μg/mL LPS for 6 h in microglia, but only at doses of 1 and 5 μg/mL. When LPS‐RS was administered alone, it did not significantly reduce TNF‐α protein levels at doses of 0.5, 1, and 5 μg/mL.^27^ In our study, when MSCs were treated with the same TLR4 antagonist, the TNF‐α gene expression showed a 0.88‐fold decrease at a dose of 1 μg/mL, a 0.61‐fold decrease at a dose of 5 μg/mL, and a 0.43‐fold decrease at a dose of 25 μg/mL. In the same study, LPS‐RS significantly reduced the cell viability of microglia by approximately 30%–40% at doses of 10 and 20 μg/mL. However, in our study, the same antagonist reduced cell viability by 4.3% in the 10 μg/mL group and 6.5% in the 100 μg/mL group. This indicates that TLR4 inactivation affects cell survival rates to different extents in different cell types.

When SKOV3 ovarian cancer cells were cocultured with MSCs, MSC1 and MSC2 cells in a direct co‐culture model for 72 h, they found that the MSC1 phenotype inhibited invasion and migration.^13^ In our study, although we did not observe the same level of antitumor effect in both MSCs and MSC1 in direct co‐culture, we observed an antiproliferative effect when the MSCs were 10 times more abundant. The difference observed in MSCs can be explained by the more aggressive nature of Panc‐1 cells compared to SKOV‐3 cells. In the same model, when MSCs were co‐cultured with SKOV3 cells, it was observed that MMP2 was secreted less compared to the condition where only SKOV3 cells were present.^18^ In contrast, in our study, we observed a decrease in MMP‐2 expression in proinflammatory andnaive MSCs in the indirect co‐culture where MSCs were 10 times more abundant. MMP2 can be considered as a regulator in these processes. In the same study, HeLa, OVCAR, SKOV3, Panc‐1, and MDA‐MB‐231 cancer cells were co‐cultured in a 3D model for 14 days to assess colony formation capacity. At the end of the experiment, the MSC1 group showed the least colony formation. The MSC1 phenotype inhibited cell growth and proliferation, thereby preventing colony formation.^18^ In our study, we did not observe an inhibitory effect on proliferation in the group where MSC1 phenotype had 10 times fewer MSCs. However, in the group where MSCs were 10 times more abundant, we observed the least cell proliferation with a proinflammatory phenotype similar to MSC1. Colony formation results were least observed in thenaive and proinflammatory groups. The similarity of the results in different proportions could be attributed to the difference in cell numbers in the co‐culture, the MSC1 phenotype not being equally proinflammatory, or the difference in co‐culture duration.

In an indirect model, PDAC‐derived Capan‐1 cells (ADMSC: Capan‐1) were co‐cultured with ADMSCs at ratios of 1:100, 1:5, and 1:1 for 48 h using an insert system. It has been reported that as the number of MSCs in the medium increased, the proliferation rate decreased, and MSCs reduced the proliferation of Capan‐1 cells by 36.5% in the 1:1 culture ratio.^28^ In our study, in the indirect model at a 1:10 ratio, we observed a 29.5% reduction in Panc‐1 cell proliferation at 48 h. This difference may be explained by the different proliferative capacities of different pancreatic cell lines. In the same study, it was shown that MSCs inhibited the Rb protein in Capan‐1 cells, arrested the cell cycle in the G1 phase, and reduced the expression of Cyclin‐dependent kinase 4 (CDK4) and cyclin D1 in vivo. Our results are in line with these findings, as we observed an increase in the number of cells in the S phase of the cell cycle and a decrease in the expression of the cyclin D1 gene. Capan‐1 cells treated with conditioned ADMSC medium slowed down the cell cycle and reduced the expression of CDK4 and cyclin D1 compared with control medium‐treated cells.^28^ Similarly, in our study, ADMSCs significantly reduced the number of cells in the G1 phase and the expression of the cyclin D1 gene, which encodes cyclin D1, in both MSC and proinflammatory MSC groups. In the same study, conditioned medium from ADMSCs induced cell death in Capan‐1 cells, especially through necrotic cell death. In our study, ADMSCs also induced cell death, but it was through apoptosis rather than necrosis.

The MSCs derived from amniotic fluid induced apoptosis by regulating the expression of apoptosis‐associated genes in a 1:1 ratio 2D and 3D co‐culture model with MiaPaCa‐2 cells.^20^ We observed the highest rate of apoptotic cells when co‐cultured with MSCs in Panc‐1 cells, indicating the proapoptotic effects of MSCs. Similarly, the expression of many genes associated with metastasis, such as MMP2, KDR, and MMP9, decreased in Panc‐1 cells co‐cultured with MSCs. Okumura et al. demonstrated that adipose‐derived stromal cells promote tumor progression by generating dense collagen matrices in the extrapancreatic tissue, leading to the densification of the tumor stroma.^29^ Similarly, we observed expression changes in the COL1A1 gene when Panc‐1 cells were co‐cultured with ADMSCs, indicating a significant role of MSCs in shaping the extracellular structures of the stroma.

In a study by Chen et al., it was revealed that human amniotic fluid‐derived MSCs suppress tumor growth in various cancers.^30^ In the relevant study, in an indirect co‐culture model, Panc‐1 cell lines were co‐cultured with MSCs in a 1:1 ratio. After 55 h, the proliferation of Panc‐1 cells was reduced by approximately 60%, and after 10 days, colony formation was reduced by 35%. After 24‐h co‐culture, the co‐cultured Panc‐1 cells showed only a 6% increase in the proportion of cells in the S phase compared to Panc‐1 cells alone. After 48 h, the co‐cultured Panc‐1 cells had an approximately 8% higher rate of apoptotic cells compared to Panc‐1 cells alone, supported by an increase in the expression of pro‐apoptotic proteins. At 40 h, migration was reduced by 26%, and at 48 h, invasion was reduced by 113%. After 48 h, the expression of several genes involved in the EMT process was affected. It was shown to decrease N‐cadherin expression by 18.2%, vimentin by 8.5%, collagen I by 30.7%, MMP‐7 by 10%, and ZEB‐1 expression by 19.5%. These findings align with our study's results. However, we could only achieve similar effects at a 10:1 ratio, which can be attributed to the different sources of MSCs and their varying anti‐tumor potentials.

Multiple myeloma cells were studied in research that demonstrated the important role of TLR4 signaling as a regulator for other cells in the proinflammatory microenvironment. In this study, it was shown that proinflammatory phenotype MSCs activate neutrophils and subsequently convert them into an immunosuppressive and pro‐angiogenic phenotype.^31^ Mathew et al. showed in an in vivo model that cancer‐associated MSCs have a high impact on promoting alternative macrophage polarization, thereby affecting tumor growth through other cells.^32^ While we have demonstrated the antitumor properties of proinflammatory MSCs against Panc‐1 cells, it is important to shed light on their effects on other cells in the microenvironment. This is because all cells in the microenvironment interact with each other and should be considered as a whole. MSCs can mediate immune activation or immunosuppression in the tumor microenvironment, so understanding their immunomodulatory functions is necessary to ensure their effectiveness against cancers. More importantly, determining the relationship between MSCs and different immune system cells and how they will affect the activity of immune cells to prevent or support tumor development is crucial. Therefore, understanding the immunomodulatory mechanisms of MSCs in cancer treatment is of great importance.^33^

We highlighted that ADMSCs were affected many different the carcinogenic molecular mechanism involved in proliferation, apoptosis, cell cycle flow, colony formation, metastasis related gene expression and immun response related cytokines. Our study is first in literature in terms of anti‐carcinogenic effects related to changing phenotypical ADMSCs in PDAC. In the light of our results, we strongly think that ADMSCs might be used as a new therapeutic approach for PDAC patients in clinic. However, the more further in vitro and in vivo investigations are need to explained detail molecular mechanisms related to cancer progression. Therefore, both further analyses are needed for the therapeutic usage as clinical trial.

## 5 CONCLUSIONS

In conclusion, the effects of MSCs can vary depending on several factors. Currently, the tumorigenic effects of MSCs are being investigated through different methods and evaluated as potential strategies. In our study, we demonstrated that ADMSCs have altered tumorigenic effects on Panc‐1 cells when stimulated via TLR4 signaling. However, this effect was altered when naive ADMSCs and ADMSCs with a pro‐inflammatory character stimulated by TLR4 agonist showed an anticarcinogenic effect on Panc‐1 cells. Anti‐inflammatory ADMSCs were showed tumor promoting effects. Understanding the role of MSCs in the tumor microenvironment will be a guiding factor for the development of microenvironment‐targeted therapeutic approaches in the future. We strongly believe that our findings will provide methodological and hypothesis‐driven benefits to both our further studies in this field and other researchers interested in the tumor microenvironment.

## AUTHOR CONTRIBUTIONS

**Demet Kaçaroğlu:** Conceptualization (equal); data curation (equal); resources (equal); software (equal); supervision (equal); validation (equal); visualization (equal); writing – original draft (equal); writing – review and editing (equal). **Seher Yaylacı:** Conceptualization (equal); investigation (equal); methodology (equal); resources (equal); visualization (equal); writing – review and editing (equal). **Nilgun Gurbuz:** Formal analysis (equal); funding acquisition (equal); project administration (equal); resources (equal); software (equal); supervision (equal); visualization (equal); writing – review and editing (equal).

## CONFLICT OF INTEREST STATEMENT

The authors declare no conflict of interest.

## ETHICS STATEMENT

Ethics approval was obtained from the Lokman Hekim University Non‐Invasive Ethics Committee with decision number 2021/136 (Code: 2012131).

## Supporting information


Appendix S1.

## Data Availability

The data that support the findings of this study are available from the corresponding author upon reasonable request.
