# Supplementary material for: Anti‐tumorigenic effects of naive and TLR4‐primed adipose‐derived mesenchymal stem cells on pancreatic ductal adenocarcinoma cells
Source: Cancer Med. 2024 Feb 1;13(2):e6964. doi: 10.1002/cam4.6964 (PMC10831913; doi:10.1002/cam4.6964)
Supplement: Supplementary file 1 — Appendix S1. [file CAM4-13-e6964-s001.docx]

**Supplementary Figure 1.**

**
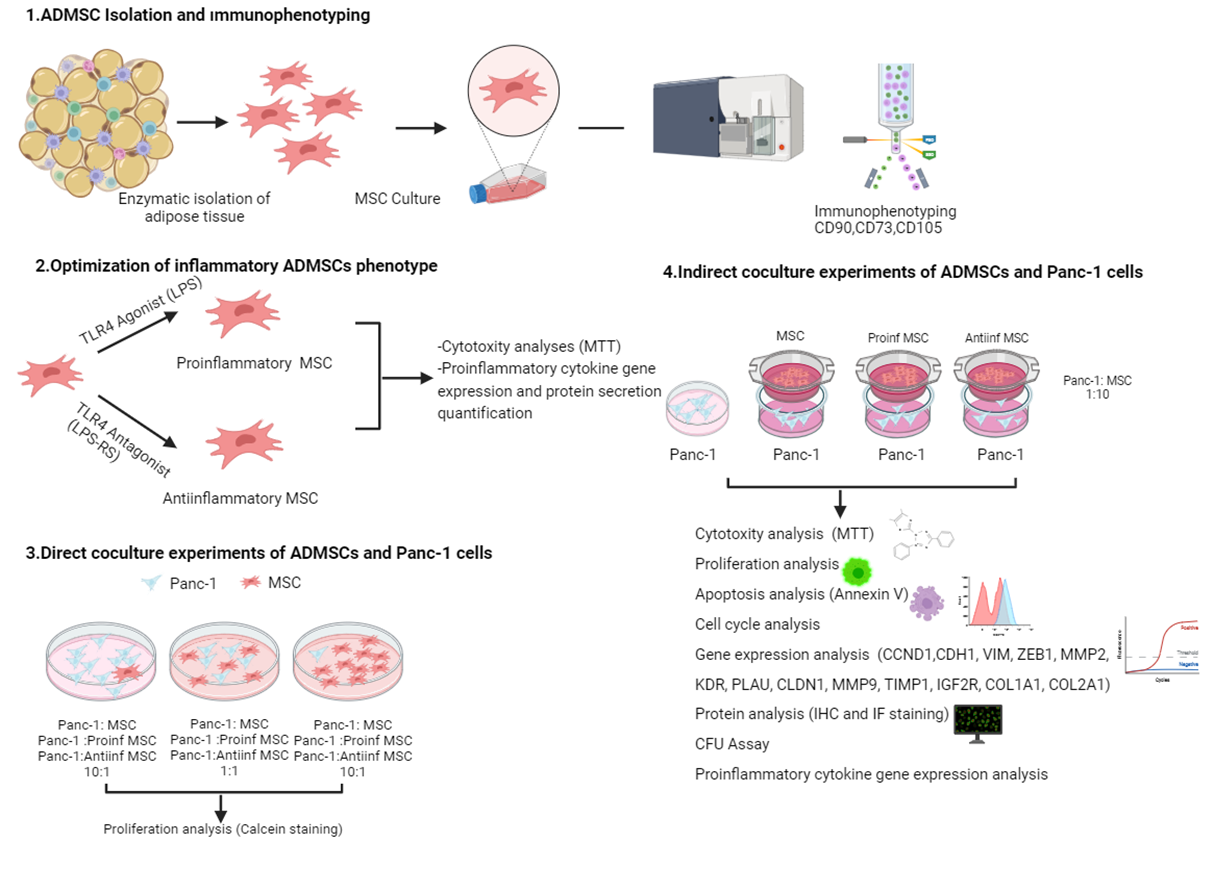
**

**Supplementary Figure 2.**


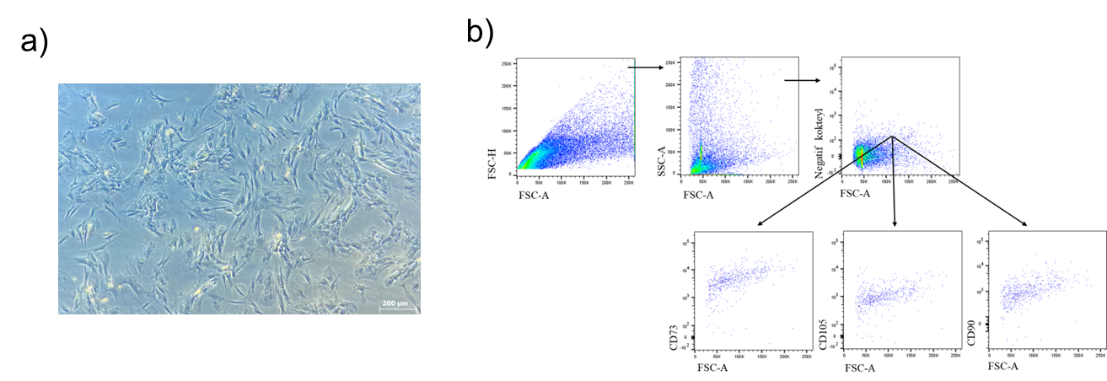


**Supplementary Figure 3.**

**
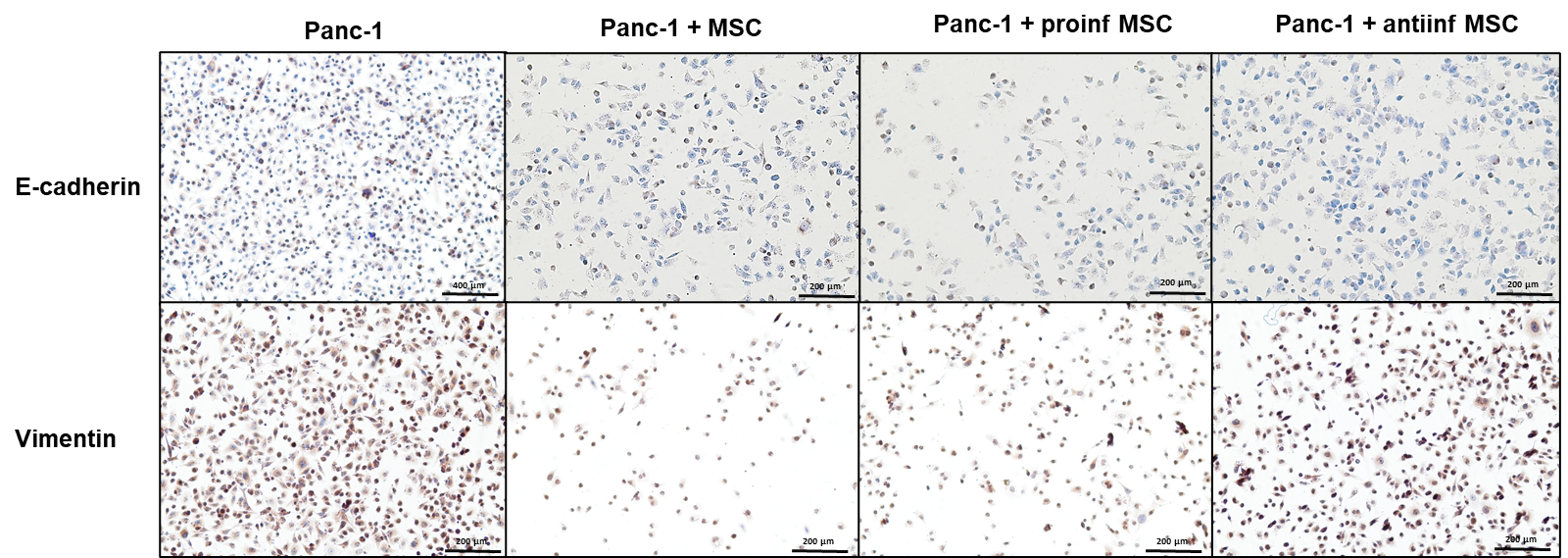
**

**Supp Table 1. RNA primers used in gene expression analysis.**

| MMP9 | F: 5′-GACGAGGGCCTGGAGTGT-3′ | R: 5′-TGTGCTGTAGGAAGCTCATCTC-3′ |
| --- | --- | --- |
| TIMP1 | F: 5′-ACCCCTGGAGCACGGCT-3′ | R: 5′-CCCACCTTCCAAGTTAGTGACA-3′ |
| GAPDH | F: 5′-GTCTCCTCTGACTTCAACAGCG-3′ | R: 5′-ACCACCCTGTTGCTGTAGCCAA-3′ |
| CCND1 | F: 5′ -CTTCCTGTCCTACTACCGCC-3′ | R: 5′-CTCCTCCTCTTCCTCCTCCT -3′ |
| IGF2R | F: 5′-CTTTGACAGCGAGAATCCCG-3′ | R: 5′- GCACTTCTTACACTTGCGGA-3′ |
| KDR | F: 5′-ATCTGTGACTTTGGCTTGGC-3′ | R: 5′-TCCCACAGCAAAACACCAAA-3′ |
| CDH1 | F: 5′-TTAGAGGTCAGCGTGTGTGA-3′ | R : 5′- CTTCTCCGCCTCCTTCTTCA -3′ |
| VIM | F: 5′-CTGCCAACCGGAACAATGAC-3′ | R : 5′- TAGTTAGCAGCTTCAACGGC-3′ |
| ZEB1 | F: 5′-AGGAGCCACAAAAGGACAGT- 3′ | R : 5′- TGGGGAATCAGAATCGTTTGC-3′ |
| CLDN1 | F: 5′- TGCTTGGAAGACGATGAGGT- 3′ | R : 5′- GAGCCTGACCAAATTCGTACC-3′ |
| COL1A1 | F: 5′- GCTACTACCGGGCTGATGAT- 3′ | R : 5′- ACCAGTCTCCATGTTGCAGA-3′ |
| MMP2 | F: 5′- TTCATTTGGCGGACTGTGAC- 3′ | R : 5′- GTGCTGGCTGAGTAGATCCA -3′ |
| IL-6 | F: 5′-CTCCACAAGCGCCTTCGGT-3′ | R : 5′-GAATCTTCTCCTGGGGGTACTGG-3′ |
| TNF-α | F: 5′-GCCCATGTTGTAGCAAACCCTC-3′ | R: 5′-GGTTATCTCTCAGCTCCACGCC-3′ |
| IL-1α | F: 5’-TGATCAGTACCTCACGGCTG-3’ | R: 5’-TGGTCTTCATCTTGGGCAGT-3’ |
| IL-1β | F: 5’-CGAATCTCCGACCACCACTA-3’ | R: 5’-AGCCTCGTTATCCCATGTGT-3’ |
| IL-8 | F: 5’-GAGAGTGATTGAGAGTGGACCAC-3’ | R: 5’-CACAACCCTCTGCACCCAGTTT-3’ |
| GM-CSF | F: 5’-AAATGTTTGACCTCCAGGAGCC-3’ | R: 5’-ATCTGGGTTGCACAGGAAGTT-3’ |
| MCP-1 | F: 5’-TGTGAACCTTTGTCTCCCCA-3’ | R: 5’-TGTGAACCTTTGTCTCCCCA-3’ |
| IFN-γ | F: 5’-GCTGTTACTGCCAGGACCC-3’ | R: 5’-TTTTCTGTCACTCTCCTCTTTCC-3’ |
